# Recall immunity limits *Litomosoides sigmodontis* growth without preventing reinfection

**DOI:** 10.3389/fimmu.2026.1961656

**Published:** 2026-10-29

**Authors:** Alexander Palmen, Lisa Marie Wadephul, Melina Hader, Severin Krayer, Kathrin Arndts, Achim Hoerauf, Manuel Ritter

**Affiliations:** 1 University of Bonn, University Hospital Bonn (UKB), Institute for Medical Microbiology, Immunology and Parasitology, Bonn, Germany; 2 University of Bonn, University Hospital Bonn (UKB), Institute for Hygiene and Public Health, GeoHealth Centre, Bonn, Germany; 3 German-West African Centre for Global Health and Pandemic Prevention (G-WAC), Partner Site Bonn, Bonn, Germany; 4 German Center for Infection Research (DZIF), Partner Site Bonn-Cologne, Bonn, Germany

**Keywords:** filarial nematodes, host-parasite interaction, *Litomosoides sigmodontis*, protective immunity, reinfection

## Abstract

**Background:**

Protective immunity against reinfection with filarial nematodes remains poorly understood. We therefore investigated whether naturally acquired immunity following spontaneous parasite clearance modifies the outcome of reinfection using the permissive murine filarial model *Litomosoides sigmodontis*.

**Methods:**

Wildtype BALB/c mice were reinfected following natural clearance of a primary *L. sigmodontis* infection and compared with mice during patent primary infection and after parasite clearance. Parasitological parameters, local immune cell responses, lung pathology, and systemic cytokine levels were assessed.

**Results:**

Adult worms were detected in only 3 of 6 reinfected mice, indicating substantial inter-individual variation in parasite establishment. Among mice with detectable adult worms, reinfection resulted in significantly shorter adult worms, while microfilaremia, embryogenesis, and *Wolbachia* density were not significantly altered, indicating restricted parasite development at the time point examined without detectable effects on parasite reproductive capacity or Wolbachia density. Reinfected mice further exhibited significantly increased neutrophil accumulation and overall immune cell infiltration into the thoracic cavity, accompanied by modestly increased lung inflammation and elevated systemic levels of IFN-γ, IL-6, IL-10, IL-22, and GM-CSF.

**Conclusion:**

Naturally acquired immunity following primary *L. sigmodontis* infection does not confer sterilising protection against reinfection, but is associated with substantial heterogeneity in parasite establishment and restricted parasite development among mice with detectable adult worms together with local and systemic recall immune responses that selectively restrict parasite growth. These findings provide new insights into naturally acquired anti-filarial immunity and the mechanisms shaping host-parasite interactions during reinfection, thereby reflecting the repeated parasite exposure that is characteristic of human filarial infections in endemic settings.

## 1 Introduction

Parasitic helminths are among the most successful pathogens, shaped by long co-evolutionary histories with their hosts and characterised by remarkable strategies to modulate host biology and immune responses (1, 2). Among these, filarial nematodes are vector-borne parasites predominantly endemic to tropical and subtropical regions. Human filarial infections are caused by several medically important species, including *Onchocerca volvulus*, the causative agent of onchocerciasis (river blindness); *Wuchereria bancrofti* and *Brugia* spp., responsible for lymphatic filariasis; *Loa loa*, which causes loiasis (African eye worm disease); and *Mansonella* spp., which cause mansonellosis. Humans can also be infected incidentally with zoonotic filarial nematodes of the genus Dirofilaria, causing human dirofilariasis. Collectively, these filarial infections affect hundreds of millions of people worldwide and represent a major public health burden in tropical and subtropical regions (3–7). Filarial infections typically induce a modified type 2 immune environment characterised by TH2 cytokines together with a strong regulatory network involving IL-10, TGF-β, IgG4, regulatory T and B cells, and alternatively activated macrophages, thereby promoting long-term parasite survival and chronic infection (8).

However, whether natural occurring filarial infection induces protective immunity against subsequent infections remains a subject of considerable debate (9, 10). Although adult filarial worms can survive for many years within the human host, several epidemiological observations suggest that naturally acquired immunity may develop in endemic populations. For example, only a proportion of individuals living in endemic areas develop patent infection or clinical disease. In addition, many infected individuals remain clinically asymptomatic despite persistent microfilaremia (the presence of circulating filarial offspring), whereas patients with chronic manifestations are frequently amicrofilaremic (3, 5, 7). Furthermore, mathematical modelling of epidemiological data has provided indirect evidence for exposure-driven herd immunity in human filariasis (11, 12). Together, these observations suggest that repeated exposure to filarial parasites may induce partial protective immunity. However, such protection appears incomplete and may decline over time. Indeed, it seems that resistance to filarial reinfections is not an all-or-nothing phenomenon but rather includes a time-dependent component, potentially reflecting short-lived immune memory responses (10). Nevertheless, direct experimental evidence demonstrating how a primary filarial infection influences the outcome of subsequent infections remains scarce.

Therefore, experimental animal models remain indispensable for dissecting the mechanisms of protective immunity, as they allow controlled primary infection, parasite clearance, and subsequent reinfections that cannot be performed in humans. The permissive rodent filarial model *Litomosoides sigmodontis* provides a unique opportunity to address this question, as primary infection can naturally resolve in immunocompetent mice (13–15), allowing subsequent reinfection under controlled experimental conditions. This enables investigation of whether immune responses generated during a primary infection provide sterilising protection, reduce parasite establishment, or instead modify parasite development and host-parasite interactions during secondary infection. Although experimental studies using *L. sigmodontis* have substantially advanced our understanding of immune-mediated parasite control, most investigations have focused on primary infection or genetically resistant hosts. These studies demonstrated that immune responses can influence parasite survival, development, and reproductive output (8, 16–18), but whether naturally acquired immunity following spontaneous parasite clearance modifies subsequent filarial infections remains largely unexplored. To address this knowledge gap, we employed the *L. sigmodontis* mouse model to determine whether naturally acquired immunity following primary infection and parasite clearance alters parasite establishment, parasite development, and local and systemic host immune responses during reinfection.

## 2 Methods

### 2.1 Mice

Female wildtype BALB/c mice (10–12 weeks old) were purchased from Jackson Laboratories (Bar Harbour, USA). Mice were kept under SPF conditions in accordance with German animal protection laws and EU guidelines 2010/63/E4 and had access to food and water *ad libitum*. All protocols and experiments were approved by the Landesamt für Verbraucherschutz und Ernährung (LAVE), Nordrhein-Westfalen, Recklinghausen, Germany (81-02.04.2018.A341, 2024-552-Grundantrag).

### 2.2 Experimental setup of *Litomosoides sigmodontis* infections

Natural infections with *Litomosoides sigmodontis* were performed via bites of infected *Ornithonyssus bacoti* mites, as previously described (15). In brief, infection in wildtype BALB/c mice leads to patency, characterised by the release of microfilariae (MF) into the peripheral blood, which peaks at approximately 70–75 days post-infection (dpi). The infection is subsequently cleared naturally by the host around 120 dpi (13, 14, 19, 20). To compare parasite survival and host immunity during patent infection, after parasite clearance, and following reinfection, three experimental mouse groups were analysed (**Figure 1**). In total, 32 mice aged 10–12 weeks were used for the experiment. Twenty mice were infected simultaneously using the same batch of infected mites and subsequently analysed at the respective experimental time points. Infected mice were assessed either at 77 dpi (time point of “patent” infection) or 158 dpi (time point of “cleared” infection). In addition, primary-infected mice, which had already cleared the infection, were reinfected at 145 days post primary infection and analysed 77 days after secondary infection (222 dpi; “reinfection”). At each corresponding time point, four age-matched uninfected naive mice were analysed in parallel as controls. Thus, naive mice were age-matched to the respective infection groups.

**Figure 1:**
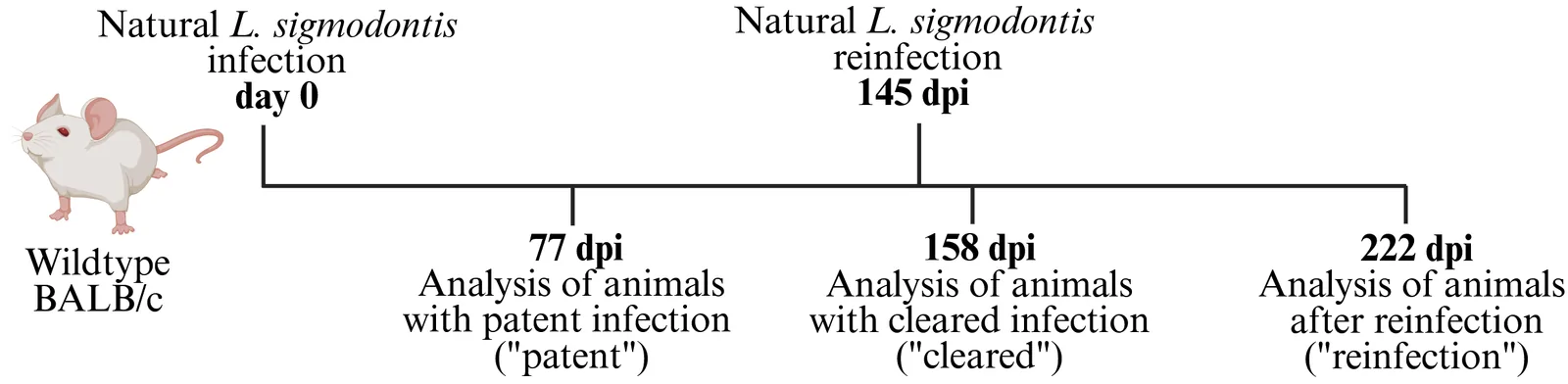
Experimental design of *Litomosoides sigmodontis* infection to assess patency, reinfection, and natural clearance. Natural infection with *Litomosoides sigmodontis* was established via bites of infected *Ornithonyssus bacoti* mites. To assess parasite survival and host immunity, three cohorts were analysed: patent infection (77 dpi), cleared infection (158 dpi), and reinfection following natural clearance (primary infection at 145 dpi, analysed 77 days after secondary infection). *L. sigmodontis*: *Litomosoides sigmodontis*; dpi: days post-infection. Created in https://BioRender.com.

### 2.3 Assessment of worm and microfilarial burden

Mice were sacrificed by inhaling Forene^®^ (Piramal Critical Care, West Drayton, UK) on day 77 dpi (patent infection), 158 dpi (cleared infection) or 222 dpi (reinfection) as previously described (18). In brief, using sterile scissors and tweezers, the abdomen and sternum were opened in the mice without damaging the diaphragm. A small opening was made at the upper border of the diaphragm, and the thoracic cavity was washed several times with sterile PBS (Thermo Fisher Scientific, Schwerte, Germany) using sterile plastic Pasteur pipettes (Ratiolab, Dreieich, Germany). The collected thoracic cavity fluid was passed through gauze to recover *L. sigmodontis* worms for assessment of worm burden, sex, and length. After separation of the worms, the thoracic cavity fluid was further analysed for immune cell composition (see section 2.6). In addition, peripheral blood was obtained from the cheek vein for systemic cytokine analysis (see section 2.7) and assessment of microfilarial (mf) counts (offspring of adult worms). In brief, 25 µl of peripheral blood was transferred into 300 µl Hinkelmann solution (Merck KGaA, Darmstadt, Germany) and incubated for 10 minutes at room temperature. Upon centrifugation for 5 minutes at 400 × g using the Eppendorf 5415 R centrifuge (Eppendorf AG, Hamburg, Germany), the sediment was microscopically assessed for the presence of mf using the Leica DM IL microscope (Leica Microsystems GmbH, Wetzlar, Germany).

### 2.4 Embryogram of female adult worms and *Wolbachia* levels

To analyse the embryonic stages, individual female worms were mechanically disrupted in 80 µl PBS (Thermo Fisher Scientific) and incubated with 20 µl Hinkelmann solution (Merck KGaA). Subsequently, 10 µl of the suspension was examined microscopically using a Leica DM IL microscope to determine the presence and number of eggs, morulae, pretzel stages, and stretched microfilariae. In addition, levels of *Wolbachia* DNA in individual female worms were quantified using a duplex PCR measuring *Wolbachia* FtsZ and actin as previously described (21).

### 2.5 Assessment of lung pathology

The lung pathology was analysed as previously described (22, 23). Briefly, lungs were placed in tissue embedding cassettes (Carl Roth, Karlsruhe, Germany), fixed in 4% formaldehyde (Carl Roth) for 24 hours, and stored in distilled water at room temperature. Dehydration was performed using a Spin Tissue Processor STP-120 (Especialidades Médicas MYR S.L., Tarragona, Spain) through a graded ethanol series (Merck, Darmstadt, Germany). Samples were then cleared with xylene (Engelbrecht, Munich, Germany) and embedded in paraffin using the HistoServe EC350 embedding station (Especialidades Médicas MYR S.L.). Sections (4-5 μm) were cut with a Rotary 3005E semi-electronic microtome (PFM Medical, Cologne, Germany). Slides were stained with haematoxylin and eosin (Carl Roth) and examined microscopically. Lung inflammation was scored from 0–4 based on immune cell infiltration towards the outside of the lung (0 = no infiltration; 1 = one to two cell layers; 2 = three to four; 3 = five to six; 4 = more than six layers or widespread infiltration).

### 2.6 Analysis of the cell composition in the thoracic cavity

To analyse the cellular composition at the site of infection (eosinophils, neutrophils, macrophages, and T cells), thoracic cavity fluid collected as described in section 2.3 was centrifuged (400 × g, 5 minutes), and the supernatant was discarded. Cells were stained with Fixable Viability Dye eFluor™ 450 (live/dead stain; Thermo Fisher Scientific) for 20 minutes at room temperature in the dark. After washing, cells were fixed and permeabilised using the eBioscience™ Foxp3/Transcription Factor Staining Buffer Set (Thermo Fisher Scientific). Fixed single-cell suspensions were subsequently cryopreserved at −20 °C in freezing medium containing 10% DMSO and 90% FCS until further analysis. For flow cytometric analysis, frozen cells were thawed, washed, and stained with fluorochrome-conjugated antibodies against surface markers for 60 minutes at 4 °C in the dark. The antibody panel consisted of CD45 BV510 (clone 30F11, BioLegend, San Diego, USA), CD11b Alexa Fluor^®^ 700 (clone M1/70, BioLegend), F4/80 BUV496 (clone T45-2342, BD Biosciences, San Jose, USA), Ly6C PerCP/Cyanine5.5 (clone HK1.4, BioLegend), Ly6G APC/Cy7 (clone 1A8, BioLegend), Siglec-F PE (clone E50-2440, BD Biosciences), CD3 BV785 (clone 17A2, BioLegend), and CD4 BUV737 (clone GK1.5, BD Biosciences). Samples were acquired on a BD LSR Fortessa flow cytometer (BD Biosciences) at the Flow Cytometry Core Facility of the University Hospital Bonn and analysed using FlowJo^©^ v10 (Treestar, Ashland, OR, USA). Compensation beads and unstained controls were used to correct for spectral overlap between fluorochromes, while fluorescence minus one (FMO) controls were included to distinguish positive from negative antigen expression. A detailed gating strategy for all analysed cell populations is provided in **Figure 2**.

**Figure 2:**
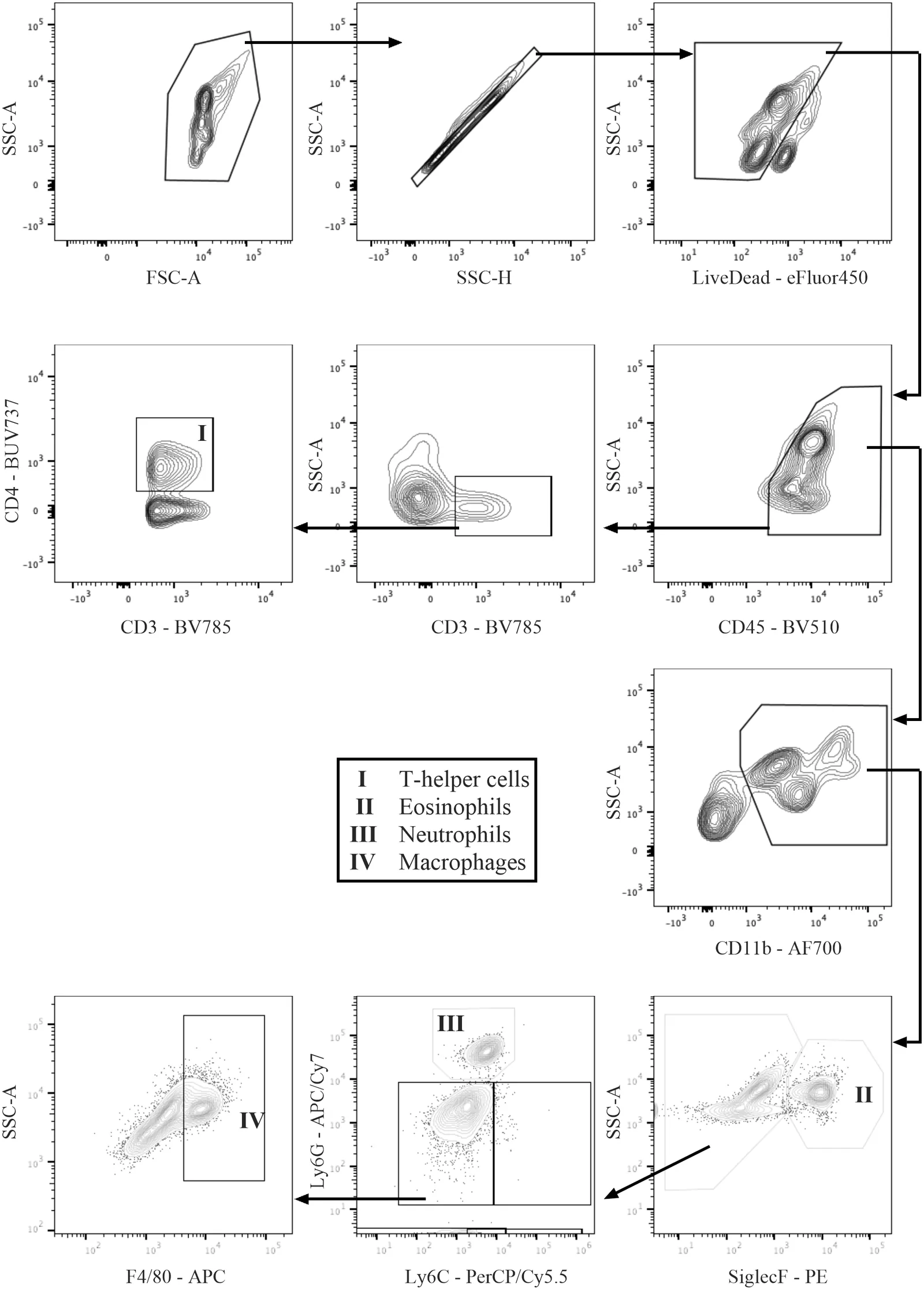
Gating strategy of T-helper cells, eosinophils, neutrophils and macrophages from the thoracic cavity. Thoracic cavity cells were initially gated based on forward scatter area (FSC-A) and side scatter area (SSC-A) to exclude debris, followed by doublet discrimination using SSC-A versus SSC-H (height). Live leukocytes were identified by exclusion of Live/Dead eFluor450-positive cells and gating on CD45-positive cells. T-helper cells were defined as CD3^+^CD4^+^ cells. Eosinophils were identified as CD11b^+^Siglec-F^+^ cells. The Siglec-F^−^ population was subsequently analysed to identify neutrophils (Ly6G^+^) and macrophages (Ly6C^−^F4/80^+^). Fluorescence-minus-one (FMO) controls were used to define positive staining and establish gating boundaries.

### 2.7 Analysis of systemic cytokine and chemokine levels

Peripheral blood (see section 2.3) was allowed to clot overnight at 4 °C, and serum was obtained by centrifugation at 1,200 × g for 10 min. Serum cytokine and chemokine levels representing TH1, TH2, TH9, TH17, TH22, and regulatory T cell responses. The following analytes were measured using the Invitrogen ProcartaPlex™ Mouse TH1/TH2/TH9/TH17/TH22/Treg 17-plex kit (Thermo Fisher Scientific) on a Luminex™ MAGPIX system (Luminex Corporation, Austin, USA), according to the manufacturer’s instructions: GM-CSF (2.4–10,000 pg/ml), IFN-γ (1.2–5000 pg/ml), IL-1β (1.2–5000 pg/ml), IL-2 (1.2–5000 pg/ml), IL-4 (1.2–5000 pg/ml), IL-5 (2.4–10,000 pg/ml), IL-6, IL-9, IL-10 (2.4–10,000 pg/ml), IL-12p70 (2.4–10,000 pg/ml), IL-13 (2.4–10,000 pg/ml), IL-17A (1.2–5000 pg/ml), IL-18 (36.6–150,000 pg/ml), IL-22 (12.2–50,000 pg/ml), IL-23 (12.2–50,000 pg/ml), IL-27 (2.4–10,000 pg/ml), and TNF-α (3.7–15,000 pg/ml).

### 2.8 Analysis of *L. sigmodontis*-specific immunoglobulin levels

For antigen-specific ELISA, 96-well plates were coated overnight at 4 °C with *L. sigmodontis* antigen (10 μg/ml in PBS, pH 9.6). After blocking with PBS containing 1% BSA, mouse sera were diluted 1:100 and incubated for 2 h at room temperature. *L. sigmodontis*-specific IgG1, IgG2a/2b, IgE, and IgM were detected using biotinylated rat anti-mouse antibodies (BD Biosciences), followed by streptavidin-HRP. After addition of the substrate, the reaction was stopped with 2M H_2_SO_4_ and absorbance was measured using a SpectraMax 190 microplate reader (Molecular Devices).

### 2.9 Statistics

Statistical analyses were performed using Prism 11 (GraphPad Software, Inc., La Jolla, USA). Normality was assessed using the D’Agostino–Pearson omnibus test. As datasets were non-normally distributed, non-parametric tests were applied. Comparisons between two groups were performed using the Mann-Whitney U test, and multiple-group comparisons were analysed using the Kruskal-Wallis test followed by Dunn’s *post hoc* test for pairwise comparisons. P values ≤ 0.05 were considered statistically significant.

## 3 Results

### 3.1 Reinfection limits *L. sigmodontis* worm growth without affecting parasite burden or reproductive capacity

To investigate how prior infection influences parasite persistence, host immunity, and the outcome of reinfection, wildtype BALB/c mice were infected with *L. sigmodontis* and subjected to secondary infection after natural clearance of the primary infection (145 dpi). As illustrated in **Figure 1**, mice were analysed during patent infection (77 dpi), after parasite clearance (159 dpi), or following reinfection (222 dpi). Throughout the experimental period, infected mice continuously gained weight, and no signs of weight loss or abnormalities in animals’ behaviour, fur and of the connective tissue were observed (**Figure 3A**), indicating the good wealth of re-infected animals. As expected, no worms were detected in animals that had cleared the infection. Therefore, no further parasitological analyses were performed in this group. Although the total worm burden did not differ significantly between mice with a primary patent infection and those undergoing reinfection, adult worms were detected in only 3 of 6 reinfected mice in the pleural cavity (**Figure 3B**). Among these three reinfected mice, both female and male worms were significantly shorter than those recovered from mice with a primary patent infection (**Figures 3C, D**). In contrast, peripheral microfilarial loads remained comparable between patent and reinfected mice (**Figure 3E**), and no differences in the distribution of embryogenic stages within female worms were detected (**Figure 3F**). Furthermore, quantitative analysis of *Wolbachia* density, assessed by the ratio of *Wolbachia* FtsZ to host actin gene copies, revealed no significant differences between patent and reinfected animals (**Figure 3G**). Together, these findings demonstrate that previous exposure to *L. sigmodontis* does not reduce parasite establishment or reproductive capacity following reinfection but selectively restricts parasite growth, resulting in shorter adult worms, suggesting that immune memory generated during primary infection appears to influence parasite development rather than parasite burden or fecundity.

**Figure 3:**
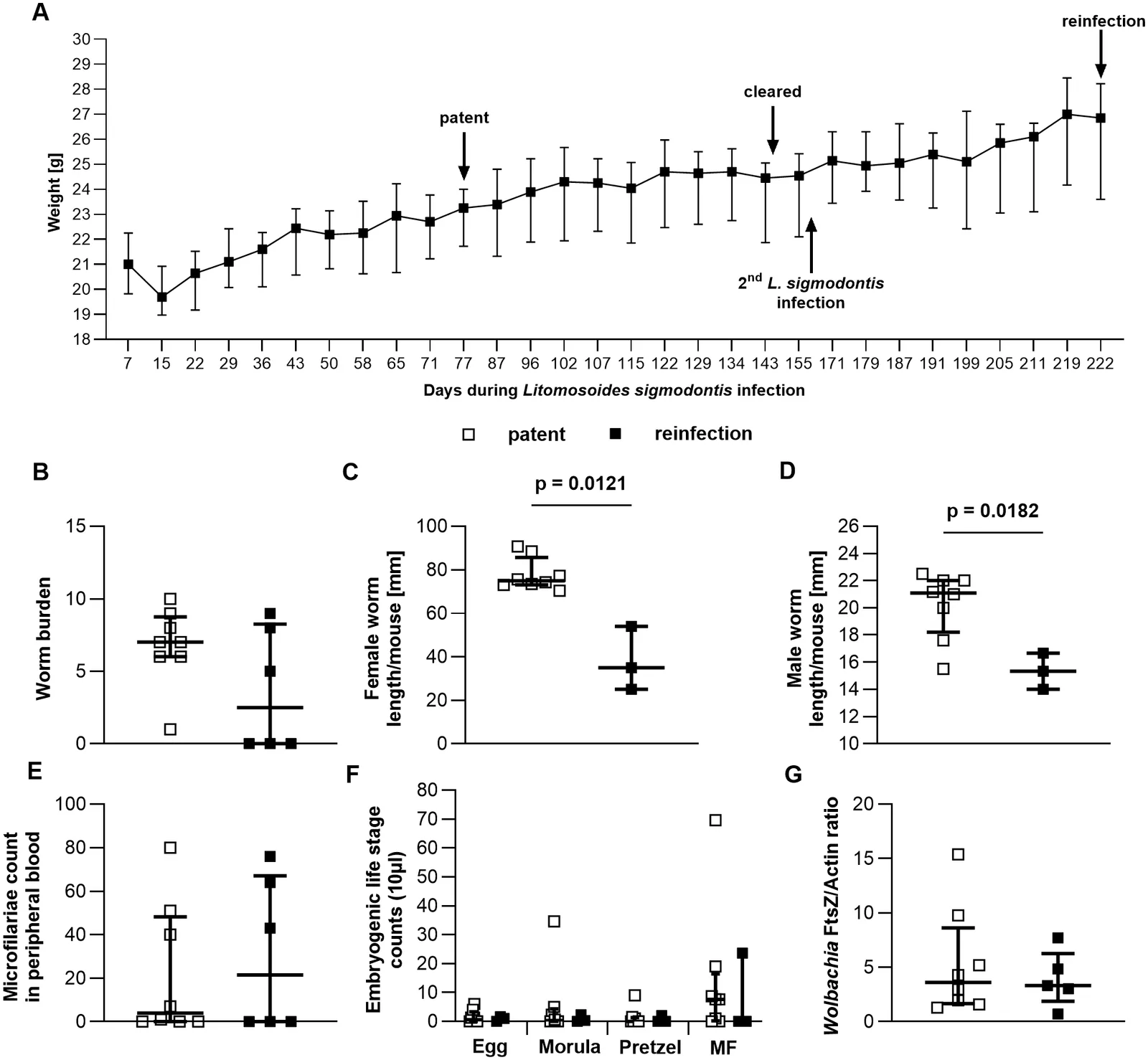
Previous infection limits *L. sigmodontis* worm growth but does not affect parasite burden or reproductive capacity. Wildtype BALB/c mice were infected with *L. sigmodontis* and reinfected after natural clearance of the primary infection (145 dpi). Mice were analysed during patent infection (77 dpi) or following reinfection (222 dpi). **(A)** Body weight development throughout the infection period. **(B)** Total adult worm burden recovered from the thoracic cavity of patent and reinfected mice. Length of female **(C)** and male **(D)** adult worms from mice in which adult worms were detected. **(E)** Peripheral microfilarial load determined in blood samples. **(F)** Quantification of embryogenic stages within female worms from mice in which adult worms were detected. **(G)** Relative *Wolbachia* density determined by quantitative PCR analysis of *Wolbachia* ftsZ gene copies normalised to host actin gene copies. Data were obtained from two independent infection experiments. Groups consisted of n = 8 mice for patent infection and n = 6 mice for reinfection experiments. For *Wolbachia* analysis **(G)**, samples were obtained from 8 female worms derived from 5 patently infected mice and 5 female worms from 2 reinfected mice. Data are presented as median and interquartile range. Statistical analysis was performed using the Mann-Whitney U test.

### 3.2 Reinfection is associated with increased immune cell infiltration into the thoracic cavity

To investigate whether the reduced worm growth observed following reinfection (**Figure 3**) was associated with enhanced local immune responses, we next characterised the cellular composition of the pleural cavity, the site of *L. sigmodontis* infection using flow cytometry. As previously reported, naïve mice exhibit only minimal immune cell infiltration into the thoracic cavity (18, 20), which was comparable to that observed in mice that had naturally cleared the primary infection. In contrast, in the present study both patently infected and reinfected mice displayed significantly higher total cell numbers in the thoracic cavity than mice that had cleared the infection. Notably, reinfected animals showed a trend toward greater cellular infiltration than patently infected mice (**Figure 4A**). To identify the immune cell populations contributing to this response, thoracic cavity cells were analysed according to the gating strategy shown in **Figure 2**. Neutrophil numbers were significantly increased in both patent and reinfected mice compared with animals that had naturally cleared the infection. Similar to the total cell counts, neutrophil numbers were higher in reinfected mice than in patently infected animals, although this difference did not reach statistical significance (**Figure 4B**). In contrast, macrophage numbers were only significantly elevated in patently infected mice and reinfected animals show similar numbers compared to mice that cleared the infection (**Figure 4C**). Neither eosinophil nor T-helper cell numbers differed significantly between the experimental groups (**Figures 4D, E**). We have previously shown that immune cell infiltration into the thoracic cavity during *L. sigmodontis* infection is associated with increased accumulation of inflammatory cell layers within the lung tissue, which can be quantified using a lung inflammation score (22). Consistent with the enhanced pleural cell infiltration observed in reinfected animals, these mice exhibited a modest increase in lung inflammation compared with the other groups (**Figure 4F**). Together, these findings indicate that reinfection elicits a more pronounced local inflammatory response, characterised primarily by increased overall cellular infiltration and a trend toward enhanced neutrophil recruitment. In combination with the reduced adult worm size observed in **Figure 3**, these results suggest that recall immune responses generated during the primary infection alter the local inflammatory milieu, thereby restricting parasite growth without reducing parasite establishment or reproductive capacity.

**Figure 4:**
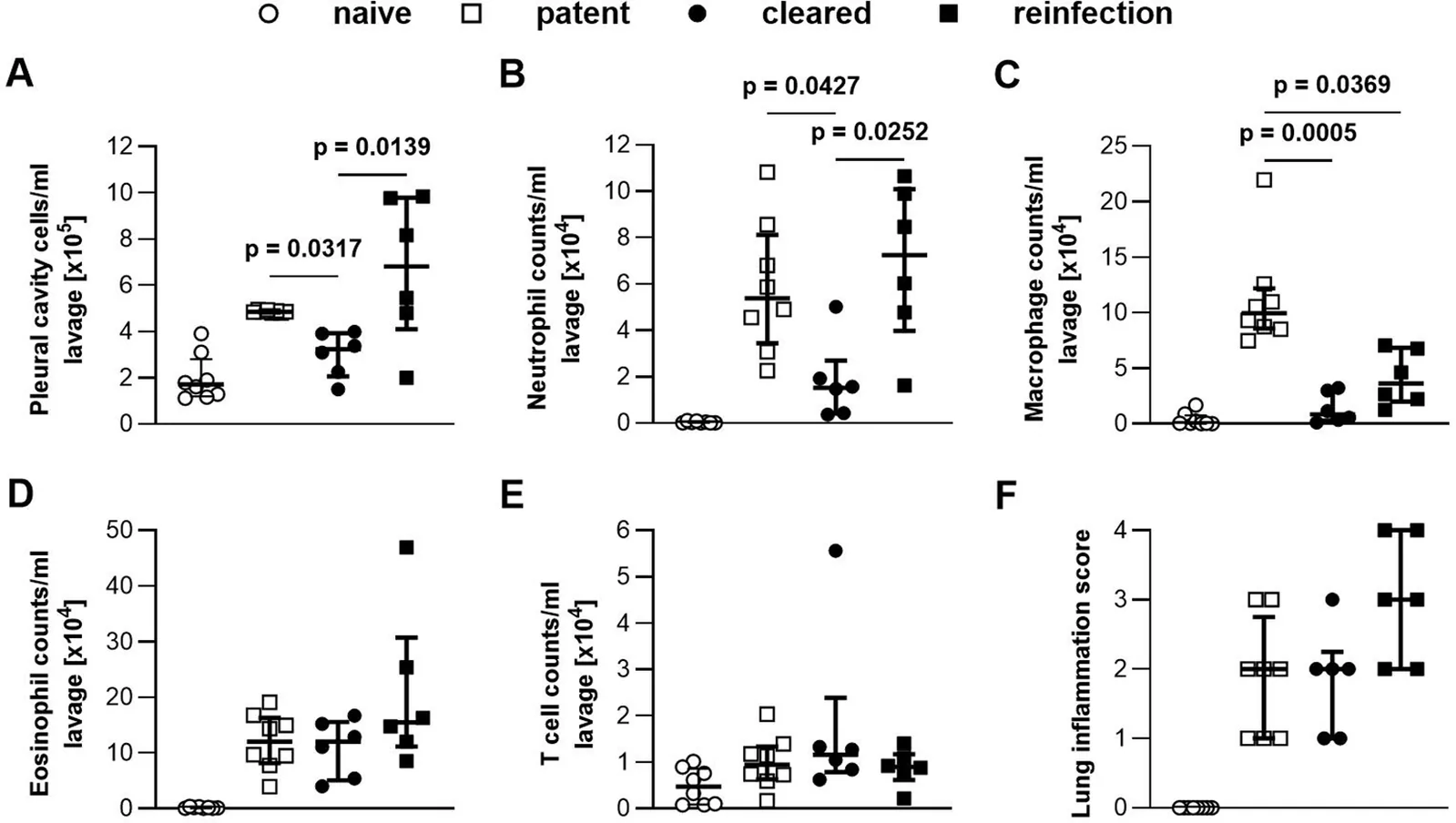
Cellular composition of the thoracic cavity following *L. sigmodontis* infection and reinfection. Wildtype BALB/c mice were infected with *L. sigmodontis* and reinfected after natural clearance of the primary infection (145 dpi). Mice were analysed after clearance of the primary infection (159 dpi), during patent infection (77 dpi), or following reinfection (222 dpi). **(A)** Total cell numbers recovered from the thoracic cavity. **(B)** Neutrophil, **(C)** macrophage, **(D)** eosinophil, and **(E)** T-helper cell counts in the thoracic cavity determined by flow cytometry according to the gating strategy shown in **Figure 2**. **(F)** Lung inflammation scores determined by histopathological analysis. Data were obtained from three independent infection experiments. Groups consisted of n = 8 naïve mice (n = 4, n = 2, and n = 2 age-matched to the patent, cleared, and reinfection groups, respectively), n = 8 mice with patent infection, n = 6 mice that had cleared the infection, and n = 6 reinfected mice. Data are presented as median and interquartile range. Statistical differences between the infection groups were performed using the Kruskal-Wallis test followed by Dunn’s multiple-comparison test.

### 3.3 Enhanced systemic cytokine, chemokine and *L. sigmodontis*-specific immunoglobulin responses following *L. sigmodontis* reinfection

To determine whether systemic immune responses contribute to the altered host-parasite interaction observed following reinfection, serum cytokine and chemokine profiles were analysed using a TH1/TH2/TH9/TH17/TH22/Treg 17-plex Luminex assay. The majority of analysed cytokines and chemokines were either below the detection limit or showed no significant differences between the experimental groups, including IL-1β, IL-2, IL-4, IL-5, IL-9, IL-13, IL-17A, IL-18, IL-23, IL-27, and TNF-α. In contrast, reinfected mice exhibited significantly increased systemic levels of IFN-γ, IL-6, IL-10, IL-22, and GM-CSF compared with patently infected animals (**Figures 5A–E**), indicating an altered systemic cytokine response following secondary infection. Furthermore, IL-12p70 levels were significantly elevated in mice that had cleared the primary infection and showed a similar tendency in reinfected mice compared with patently infected animals (**Figure 5F**).

**Figure 5:**
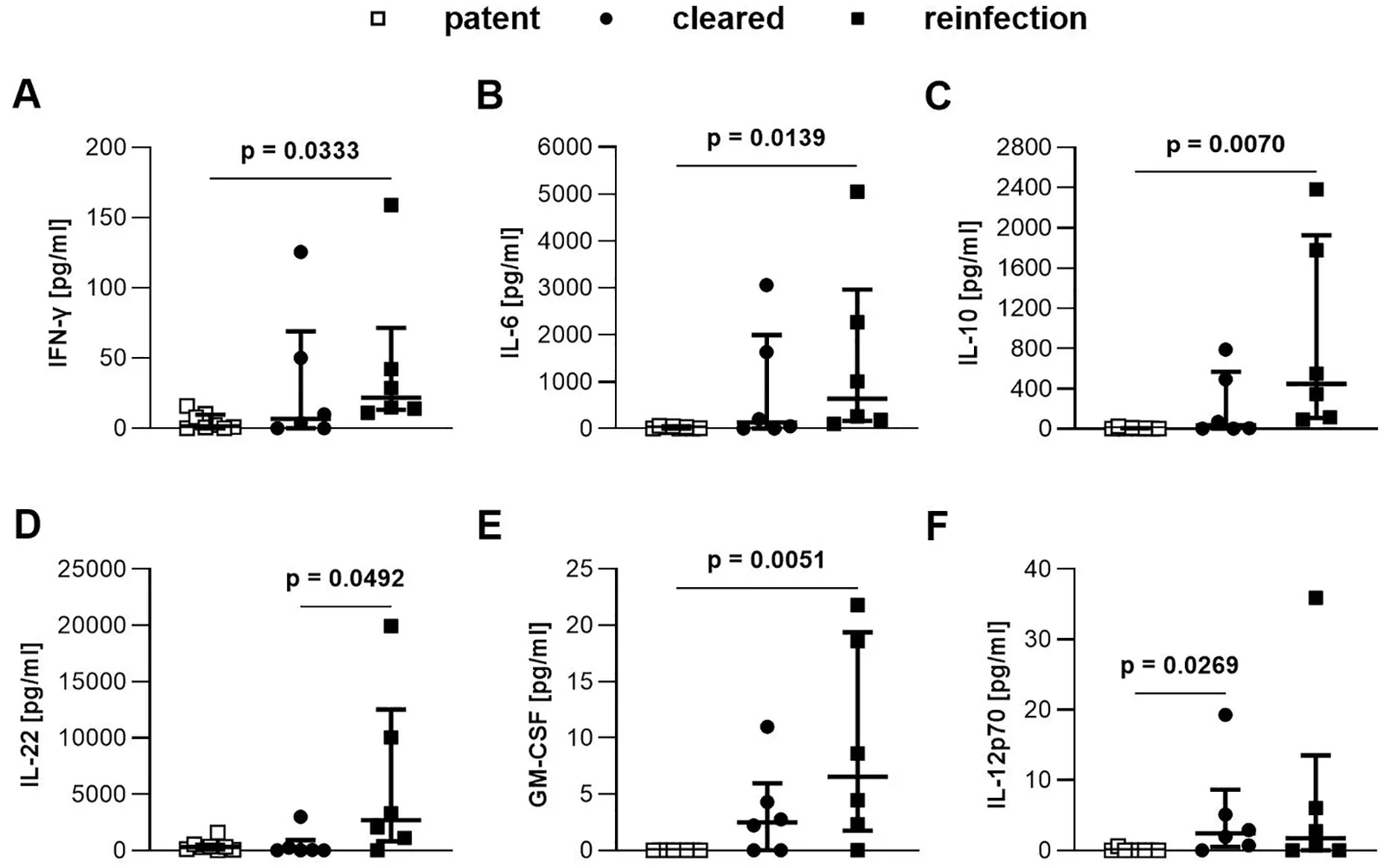
Increased systemic cytokine and chemokine levels following *L. sigmodontis* reinfection. Serum levels of IFN-γ **(A)**, IL-6 **(B)**, IL-10 **(C)**, IL-22 **(D)**, GM-CSF **(E)**, and IL-12p70 **(F)** were quantified using a Luminex assay. Cytokine and chemokine levels of infected groups were normalised by subtracting the corresponding values obtained from naïve control mice (n = 4 naïve mice per infection group). Data were obtained from three independent infection experiments. Groups consisted of n = 8 mice for patent infection, n = 6 mice for cleared infection, and n = 6 mice for reinfection experiments. Data are presented as median and interquartile range. Statistical analysis was performed using the Kruskal-Wallis test followed by Dunn’s multiple-comparison test.

To further characterise systemic immune responses following reinfection, *L. sigmodontis*-specific immunoglobulin levels were analysed by ELISA. Reinfected mice exhibited significantly increased IgG1 levels compared with patently infected mice (**Figure 6A**). IgG2a/2b and IgE levels were also higher in reinfected mice, although these differences did not reach statistical significance (**Figures 6B, C**). IgM levels did not differ between patently infected and reinfected mice but were slightly reduced in mice that had cleared the primary infection (**Figure 6D**). In mice that had cleared the primary infection, IgG1, IgG2a/2b, and IgE levels were also higher than in patently infected mice, although these differences did not reach statistical significance. The complete measurements for all analysed cytokines, chemokines, and *L. sigmodontis*-specific immunoglobulins, including values from naïve controls, are provided in the **Supplementary Table**.

**Figure 6:**
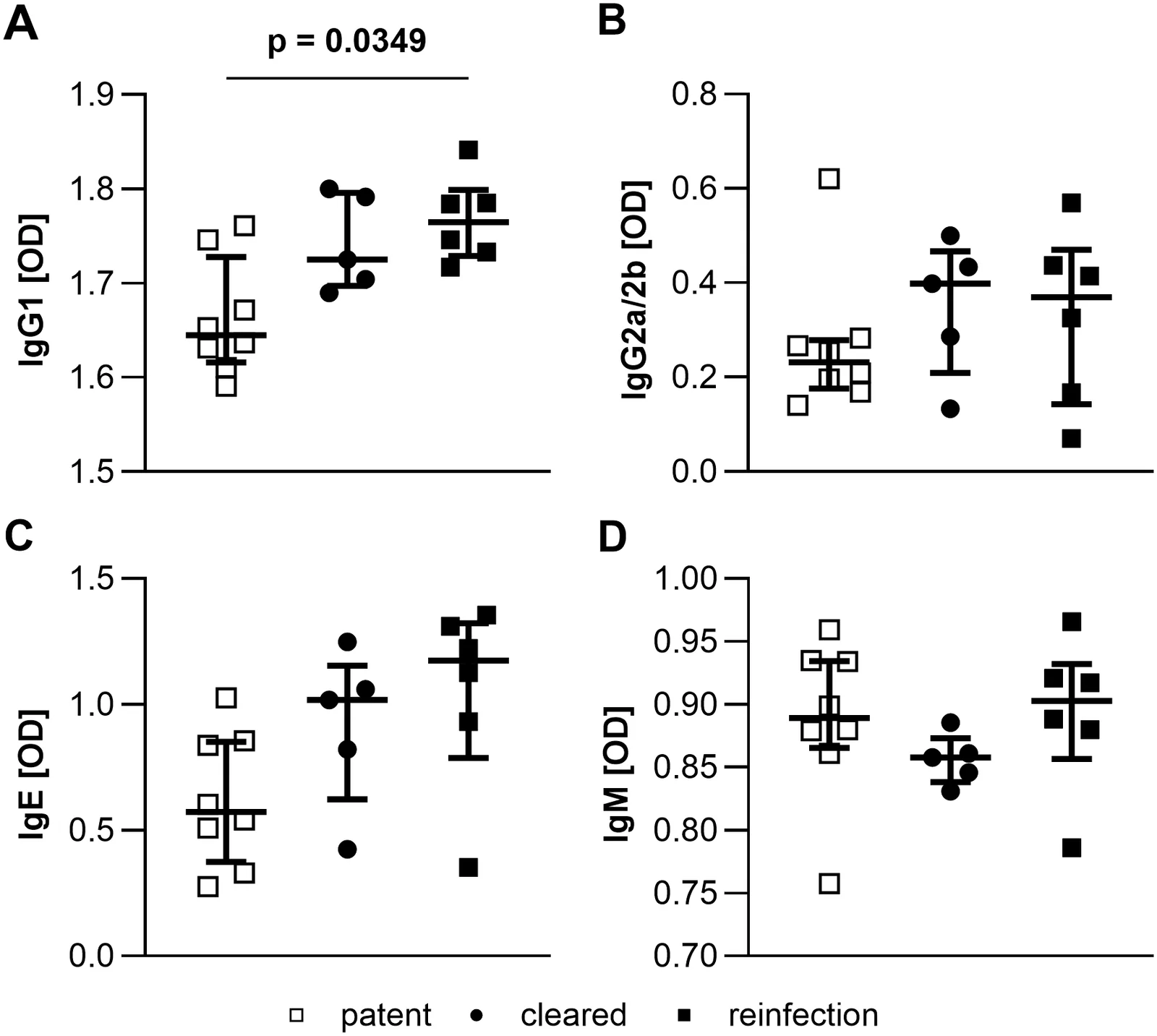
Altered *L. sigmodontis*-specific antibody levels in the reinfection groups. Serum levels of **(L)** sigmodontis-specific IgG1 **(A)**, IgG2a/2b **(B)**, IgE **(C)**, and IgM **(D)** were quantified using the ELISA technique. Antibody levels are expressed as optical density (OD), reflecting the amount of antigen-specific antibody detected, and were normalised by subtracting the corresponding mean OD values of naïve control mice (n = 4 per infection group). Data were obtained from three independent infection experiments. Groups consisted of n = 8 mice for patent infection, n = 5 mice for cleared infection, and n = 6 mice for reinfection experiments. Data are presented as median and interquartile range. Statistical analysis was performed using the Kruskal-Wallis test followed by Dunn’s multiple-comparison test.

Together with the increased immune-cell infiltration observed at the site of infection (**Figure 4**), these findings demonstrate that reinfection is associated with an altered immune response involving local cellular recruitment and systemic cytokine, chemokine, and *L. sigmodontis*-specific immunoglobulin responses. Notably, despite this altered immune response, reinfection did not result in a significant reduction in total adult worm burden or reproductive capacity but was associated with heterogeneous parasite establishment and restricted parasite development at the time point examined (**Figure 3**). These findings suggest that naturally acquired immune responses following primary infection may influence parasite establishment and subsequent development without providing sterilising protection.

## 4 Discussion

The present study addressed the long-standing question of whether a naturally resolved primary filarial infection confers protective immunity against subsequent reinfection. Using the permissive *L. sigmodontis* mouse model, we demonstrate that naturally acquired immunity following spontaneous parasite clearance does not provide sterilising protection or reduce parasite establishment upon secondary infection. Instead, reinfection was associated with restricted parasite growth despite unchanged parasite burden, microfilarial production, embryogenesis, and *Wolbachia* density. This altered parasite phenotype coincided with enhanced local immune cell infiltration into the thoracic cavity, increased neutrophil accumulation, and augmented systemic cytokine responses, consistent with an altered immune response following previous infection that may contribute to modifying the host-parasite interaction without preventing reinfection. Together, our findings suggest that naturally acquired anti-filarial immunity can influence parasite development and local immune activation rather than induce sterilising immunity, although the magnitude of these effects varies between individual animals.

These findings provide experimental support for epidemiological observations in human filariasis suggesting that repeated exposure can induce partial, rather than sterilising, immunity (10–12). Although evidence for naturally acquired protection in humans remains indirect, our data indicate that immune memory generated during primary infection may modify parasite biology without completely preventing subsequent infection. Thus, similar to observations in endemic populations, acquired immunity may shape parasite fitness and infection dynamics without eliminating susceptibility to reinfection. Indeed, partial acquired immunity following repeated helminth exposure has been associated with reduced parasite establishment and worm burden (24–29) and, less frequently, with reduced parasite size or restricted parasite development (30, 31).

Interestingly, the enhanced cellular infiltration observed at the site of infection in reinfected animals was primarily characterised by increased neutrophil accumulation. Neutrophils are increasingly recognised as important mediators of protective immunity against filarial nematodes and contribute particularly to the early control of invading L3 larvae. In the *L. sigmodontis* model, neutrophils have been shown to reduce parasite establishment through oxidative burst responses and the formation of neutrophil extracellular traps (NETs) (32). Similar NET-mediated neutrophil responses have been described in *Onchocerca volvulus* infection, where *Wolbachia*, the endosymbiont of most filarial species including *L. sigmodontis*, induces neutrophil activation that contributes to inflammatory responses surrounding adult worms and to the development of ocular pathology (33–36). Likewise, deficiency of the neutrophil-derived alarmins S100A8 and S100A9, which together form the inflammatory mediator calprotectin, resulted in enhanced neutrophil activation and recruitment and was associated with reduced worm burdens, suggesting that stronger neutrophil-driven inflammatory responses impair larval migration and increase resistance to infection (37). Similarly, impaired neutrophil recruitment in NOD2-deficient mice or antibody-mediated neutrophil depletion increased worm recovery, further supporting a crucial role for neutrophils in limiting filarial infection establishment (38). Together, these studies highlight the important role of neutrophils as a first line of defence against invading infective larvae. The observed increase in neutrophil numbers following reinfection, although not statistically significant, may nevertheless be relevant in the context of the heterogeneous parasite establishment observed following reinfection. Nevertheless, adult worm numbers showed a trend towards reduction following reinfection, and notably, no adult worms could be detected in half of the reinfected mice. These findings raise the possibility that immune responses generated during the primary infection may contribute to the control of invading L3 larvae and subsequent parasite establishment. In addition to the apparent reduction in parasite establishment, adult worms in reinfected mice that developed detectable adult worms were significantly shorter, indicating that parasites surviving reinfection exhibited restricted subsequent growth. This finding indicates that the altered immune environment following reinfection may extend beyond the early phase of infection and be associated with restricted parasite development after establishment. Neutrophils could potentially contribute to such an effect through oxidative mediators, NETs, or other inflammatory mechanisms acting within the local environment surrounding developing or adult worms. However, given that the difference in neutrophil numbers between reinfected and patently infected mice was not statistically significant, our data do not establish a direct causal role for neutrophils in the reduced worm growth. Rather, they raise the possibility that neutrophils may contribute to the immune response following reinfection at different stages of reinfection, with effects on both parasite establishment and subsequent development. Targeted depletion or functional studies will be required to determine whether neutrophils directly mediate these effects.

Interestingly, macrophage infiltration was significantly increased in patently infected mice compared with mice that had cleared the infection and reinfected animals. Future studies should further characterise the phenotype of the infiltrating macrophages, as alternatively activated macrophages (AAMs) are known to play a pivotal role in establishing the anti-inflammatory environment that promotes filarial survival (39–41). It will therefore be of particular interest to determine whether the reduced macrophage accumulation observed at the site of infection in reinfected mice reflects a decrease in AAMs. A reduced AAM response could potentially shift the local immune environment towards enhanced inflammatory activation and contribute to the impaired parasite growth observed following reinfection.

In addition to the enhanced local immune cell infiltration, reinfected mice exhibited significantly increased systemic levels of IFN-γ, IL-6, IL-10, IL-22, and GM-CSF. In contrast, classical TH2 cytokines, including IL-4, IL-5, and IL-13, were not altered systemically, suggesting that reinfection does not simply amplify the type 2 immune environment typically associated with chronic filarial infection, but rather elicits a broader systemic altered immune response. Importantly, however, these systemic cytokine and chemokine measurements do not allow us to determine whether the observed response is driven by antigen-specific T-cell activation or reflects a more general inflammatory response associated with reinfection. Nevertheless, the selective increase in IFN-γ, IL-6, IL-10, IL-22, and GM-CSF, together with the enhanced cellular infiltration and increased neutrophil accumulation in the thoracic cavity, is consistent with an altered immune response following previous infection. This response may reflect a combination of antigen-specific recall mechanisms and broader inflammatory responses triggered by reinfection. Previous experimental work provides evidence that infection-experienced CD4^+^ T cells can contribute to protective immunity against *L. sigmodontis*. Adoptive transfer of CD4^+^ T cells from infected mice reduced adult worm numbers and resulted in shorter remaining worms, demonstrating that CD4^+^ T cells can affect both parasite survival and subsequent parasite development. Importantly, CD4^+^ T cells from mice that had naturally cleared a primary infection retained anti-filarial activity following transfer, supporting the persistence of functional CD4^+^ T-cell-mediated immunity after parasite clearance (18). These findings provide a potential cellular basis for the altered host response observed following reinfection in our study.

For example, IFN-γ has consistently been associated with protective anti-filarial immunity during *L. sigmodontis* infection, where IFN-γ deficiency results in impaired neutrophil function, reduced inflammatory responses around adult worms, and prolonged parasite survival (42). Similarly, in human lymphatic filariasis, enhanced antigen-specific IFN-γ responses have been described in putatively immune individuals compared with microfilaremic individuals, suggesting that TH1-associated responses contribute to parasite control (43, 44). Interestingly, reinfection induced increased systemic IL-10 levels, suggesting that regulatory mechanisms remain active during secondary infection. Given the established role of IL-10-producing regulatory T cells, alternatively activated macrophages, and other regulatory populations in maintaining immune homeostasis during chronic filarial infections, persistent IL-10 responses may contribute to balancing parasite-directed inflammation and tissue damage (5, 45). However, this regulatory environment may also limit the development of highly effective anti-parasitic responses, potentially contributing to the heterogeneous parasite establishment observed following reinfection and the restricted parasite development among mice in which adult worms were detected. In addition to cellular immunity, humoral immune responses may also contribute to the altered host-parasite interaction following reinfection. In the present study, reinfected mice exhibited significantly increased *L. sigmodontis*-specific IgG1 levels compared with patently infected mice, while IgG2a/2b and IgE levels showed similar, although non-significant, increases. These findings suggest that systemic antibody responses may contribute to the heterogeneous parasite establishment observed following reinfection and the restricted parasite development among mice in which adult worms were detected. However, we did not assess *L. sigmodontis*-specific antibodies locally in the thoracic cavity and therefore cannot determine whether local antibody responses contribute to the observed phenotype. Consistent with the persistence of humoral responses observed here, recent work in the *L. sigmodontis* model has demonstrated sustained adaptive immune responses, characterised by prolonged T follicular helper (Tfh) cell and germinal-centre B-cell responses and persistent antibody production following infection, including after parasite clearance (46). These findings provide a potential mechanistic context for the antibody responses observed following reinfection and support the possibility that infection-induced humoral immunity persists after parasite clearance and may shape the immune environment upon subsequent exposure.

An important limitation of the present study is that the increased inflammatory cell infiltration following reinfection cannot be definitively attributed to an accelerated memory-driven recall response, as an age-matched primary infection group at the time of reinfection was not included. Such an additional group was not approved by the animal ethics authorities. Importantly, immune cell infiltration into the pleural cavity is closely associated with ongoing *L. sigmodontis* infection, making age-matched naive mice unsuitable as a direct control for the infection-associated cellular compartment. Nevertheless, age-matched naive controls analysed by flow cytometry at the patent and reinfection time points showed no significant differences in pleural immune cell composition. We therefore interpret the increased cellular infiltration and neutrophil accumulation as an altered inflammatory response associated with reinfection rather than as definitive evidence of an accelerated recall response. Potential effects of age on parasite development were also considered. Babayan et al. found no significant difference in adult *L. sigmodontis* survival between younger and older BALB/c mice infected with the same parasite dose, but reported significantly higher microfilarial densities in older mice (47), indicating increased parasite fecundity and suggesting that infection may be at least as successful, if not more successful, in older mice. Similarly, another study reported no relevant differences in worm length between different infection time points (40). In our ongoing age-dependent studies, we likewise observed no significant differences in adult worm burden or worm length between younger and older mice, with a tendency towards higher adult worm numbers in older animals (manuscript in revision). Together, these findings do not support host age as an explanation for the reduced parasite establishment or restricted worm development observed following reinfection. Finally, the experimental design was intended to model an important feature of natural filarial exposure, in which individuals in endemic settings may experience repeated exposure to infective vectors over many years and may be exposed again following parasite clearance or treatment. Comparing primary infection with infection after resolution therefore allowed us to investigate how previous infection influences the outcome of subsequent exposure.

The increased levels of IFN-γ and IL-22 may further contribute to the enhanced neutrophil response observed after reinfection, providing a potential link between adaptive recall and local innate effector mechanisms. While IFN-γ is not considered a primary neutrophil-recruiting cytokine, it can promote neutrophil activation, priming, and functional responses during inflammatory conditions (48, 49). In contrast, IL-22 contributes to local innate immune activation by stimulating epithelial cells to produce neutrophil-attracting chemokines, including CXCL1 and CXCL2, which can promote neutrophil recruitment at sites of inflammation (50, 51). Thus, elevated IFN-γ and IL-22 responses during reinfection may contribute to shaping a local inflammatory environment that favours increased neutrophil accumulation without necessarily resulting in enhanced parasite clearance. Although the functional significance of elevated IL-6, IL-22, and GM-CSF levels during reinfection remains to be determined, IL-6 has previously been implicated in protective immunity against *L. sigmodontis* by promoting early immune responses that restrict parasite migration and establishment. IL-6-deficient mice exhibit increased worm recovery during early infection, highlighting its contribution to innate immune-mediated control of invading larvae (52). In addition, IL-22 and GM-CSF have been associated with tissue protection, immune regulation, and myeloid cell activation, respectively (53, 54), suggesting that their increased levels during reinfection may contribute to the enhanced local immune activation observed in reinfected mice rather than directly mediating parasite clearance (53, 54). Thus, the increased IL-6, IL-22 and GM-CSF levels observed in reinfected mice, together with enhanced neutrophil accumulation and overall cellular infiltration in the thoracic cavity, are consistent with an altered local and systemic inflammatory response following reinfection. Together, these findings support a model in which durable adaptive responses, including infection-experienced CD4^+^ T cells and persistent humoral immunity, may shape the local innate response upon reinfection, potentially involving enhanced neutrophil activation and thereby restricting parasite development. However, the relative contribution of these mechanisms remains to be determined.

Collectively, this study demonstrates that naturally acquired immunity following resolved filarial infection does not prevent reinfection but alters the subsequent host-parasite interaction by restricting parasite development. These findings suggest that naturally acquired anti-filarial immunity may modulate parasite development and local immune activation rather than induce sterilising immunity, providing new insights into how repeated exposure may shape filarial infection outcomes in endemic settings.

## Data Availability

The original contributions presented in the study are included in the article/**Supplementary Material**. Further inquiries can be directed to the corresponding author.
